# Estimated need for anthelminthic medicines to control soil-transmitted helminthiases in school-aged children, 2020–2030

**DOI:** 10.1186/s40249-020-00656-9

**Published:** 2020-05-07

**Authors:** Chiara Marocco, Fabrizio Tediosi, Mathieu Bangert, Denise Mupfasoni, Antonio Montresor

**Affiliations:** 1 Department of Control of Neglected Tropical Diseases, World Health Organization, Geneva, Switzerland; 2 University of Bologna, Bologna, Italy

**Keywords:** Preventive chemotherapy, Soil-transmitted helminthiases, Drug donations

## Abstract

**Background:**

Soil-transmitted helminthiases (STH) are part of the group of neglected tropical diseases traditionally treated with preventive chemotherapy interventions. In recent years, drug donations have been essential to expanding preventive chemotherapy and achieving progressive control of morbidity from STH. This study aims to evaluate the need for anthelminthic medicines during 2020–2030.

**Methods:**

To estimate the need for anthelminthic medicines, we considered three different scenarios: (1) the control programmes continues to expand coverage and maintains the frequency of drug administration established at baseline; (2) the programmes continues to expand coverage but adapts the frequency of drug administration when the STH prevalence is reduced and (3) the STH programme becomes self-sustainable in some endemic countries.

**Results:**

We estimate that the number of anthelmintic medicines needed to treat school-aged children will increase by 40% by 2025 and by 52% by 2030 if countries do not change the frequency of preventive chemotherapy (scenario 1); that the number of tablets needed will reduce by 32.4% by 2025 and by 49.1% in 2030 if endemic countries reduce the frequency of preventive chemotherapy (scenario 2); and drug donations could be reduced by 54.4% by 2025 and 74.4% by 2030 if some endemic countries could become independent in drug procurement (scenario 3).

**Conclusions:**

The number of anthelmintic medicines needed to achieve elimination of morbidity due to STH in school-aged children will decline during 2020–2030. The decline will be substantial if a number of “upper-middle income” countries in which STH are endemic procure, as expected, anthelminthic medicines independently.

## Background

Soil-transmitted helminthiases (STH) are part of a group of diseases commonly referred to as neglected tropical diseases (NTDs) that disproportionately affect impoverished populations living in areas where financial resources and access to sanitation are limited [1]. STH affect approximately one billion people worldwide [2] and can trigger important health consequences such as severe anaemia and hamper the cognitive and physical development of children [1].

**Fig. 1:**
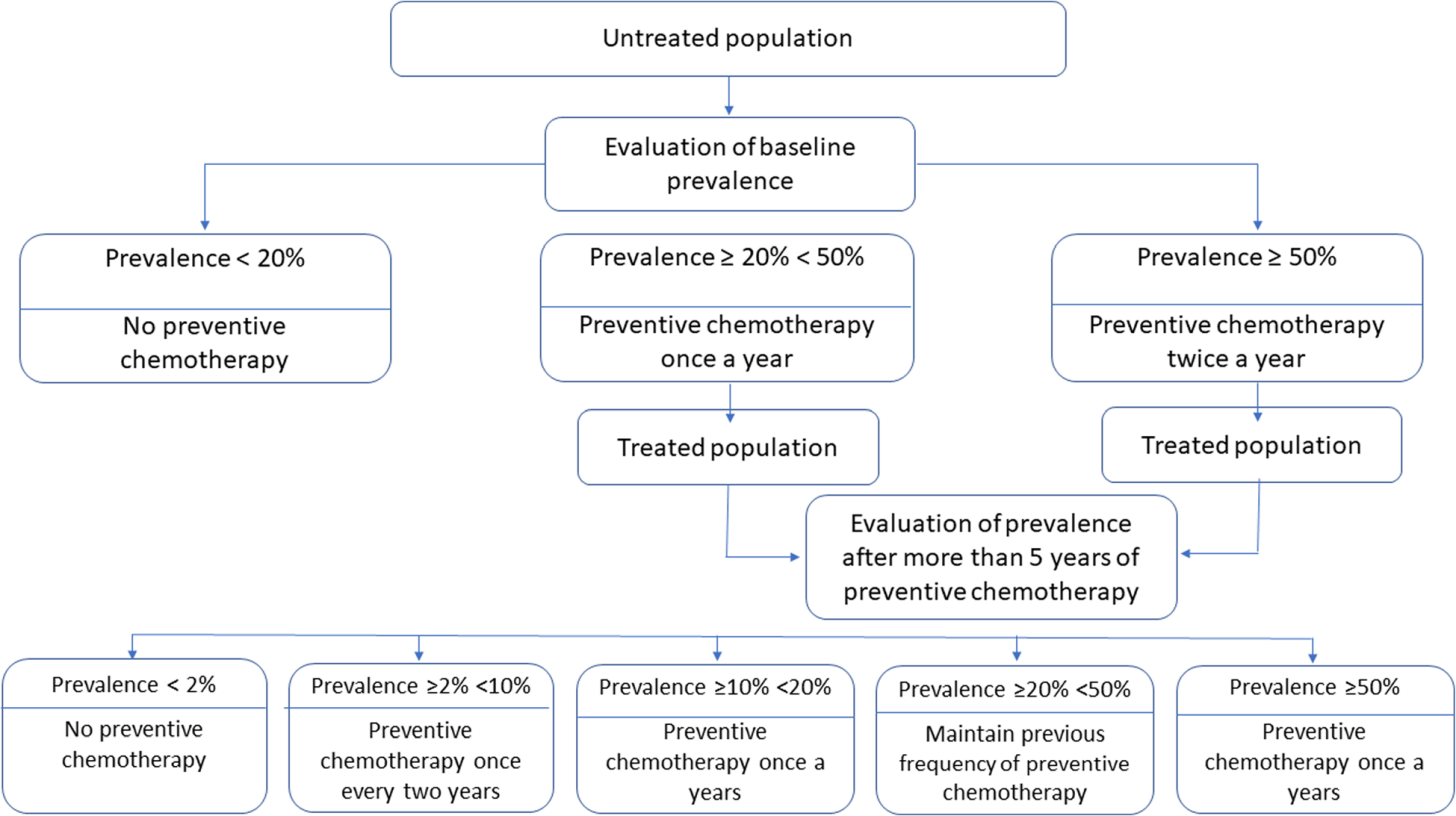
World Health Organization decision-tree (from WHO 2011). STH: Soil-transmitted helminthiases

Preventive chemotherapy (PC), the large-scale administration of anthelminthic medicines to populations at risk, is the preferred public health intervention for treatment of four NTDs: lymphatic filariasis, schistosomiasis, onchocerciasis and STH [3]. For STH, the target is, by 2020, to eliminate morbidity by reducing the prevalence of infections of heavy and moderate intensity to below 2% among school-aged children (SAC) and preschool-aged children [1]. Because the reservoir of infection in the environment is perpetuated by inefficient sanitation [3], the World Health Organization (WHO) recommends that endemic countries maintain PC interventions after morbidity is eliminated, improve coverage of sanitation to maintain the benefits of PC, evaluate the epidemiological situation of STH after five consecutive years of PC and use the decision-tree (Fig. 1) to select the optimal frequency of PC according to the epidemiological situation resulting from control activities [4].

WHO has promoted the use of PC for treatment of STH since 2003. Some countries have implemented several rounds of PC since then and have achieved high levels of coverage and reduced prevalence and intensity of STH infection [5], enabling them to reduce the need for anthelminthic medicines to maintain control of STH [6]. Albendazole and mebendazole, the medicines required by PC programmes for treatment of STH, are currently donated by GlaxoSmithKline and Johnson & Johnson to WHO for distribution to health ministries in participating endemic countries.

Scholars have identified the potential negative long-term impact on public health when donations are discontinued as well as the disincentives for generic manufacturers to enter the market for anthelminthics [7]. For this reason, the maintenance of public health interventions after discontinuation of drug donations are a concern. This study addressed two related research questions:

1. What is the global need for anthelminthic medicines for PC during 2020–2030? (Given that many countries in which STH are endemic are expected to implement progressively five consecutive years of PC with effective coverage and to reduce the frequency of the intervention); and.
2. To what extent will the need for drug donations be reduced? (Given that many countries in which STH are endemic are expected to reach a level of development that will facilitate national support of PC interventions for treatment of STH).

The first part of our hypothesis is that by 2030, the quantities of anthelminthics needed globally will reduce because of the impact of PC programmes on the prevalence of STH infection. The second part of our hypothesis is that some endemic country will progressively assume financial responsibility for deworming programmes.

## Methods

We conducted our study in several phases. First, we estimated for all countries in which STH are endemic the expected dates for achieving the 75% coverage target and the year in which all countries would complete five consecutive years of PC with effective coverage. Secondly, we estimated the number of children expected to be treated annually in each endemic country and the number of tablets needed annually until completion of five rounds of PC. Finally, we estimated which countries would be able to conduct PC independently. The software for statistical analysis R (https://www.r-project.org/) was used to analyse the data.

### Estimated year in which each endemic country will reach the coverage target

The WHO PCT databank [8], an online WHO dataset, contains information for all countries in which STH are endemic on the number of children needing PC and the annual rate of coverage since 2006. Each endemic country has followed a unique path for implementing STH control programmes, and PC has started at different times and achieved different levels of coverage. First, we extracted from the PCT databank the countries that had already reached effective coverage (i.e. 75% national coverage). Countries that had implemented three or more years of PC with effective coverage from 2011 to 2017 belonged to category A. The remaining endemic countries were divided into three subgroups, according to their mean levels of coverage during the seven years 2011–2017, in order to capture the level of effective coverage in each country in 2017. Countries with a mean coverage level of 50–75% were classified as category B (effective coverage not reached and high coverage level). Countries with a mean coverage level of 20–50% were classified as category C (effective coverage not reached and average coverage level). Countries with a mean coverage level of 0–20% were placed in category D (effective coverage not reached and low coverage).

The three categories were then attributed different expectations on how long it would take each to reach the 75% coverage target: for each endemic country in category B, we assigned the regional average time minus the standard deviation (*SD*), for each endemic country in category C, we assigned the regional average time and for each endemic country in category D we assigned the regional average plus the *SD*. The *SD* was added or subtracted to the projected number of years as a proxy for the progress countries had made until 2017 in increasing their coverage rates. Table 1 summarizes this model.

**Table 1:** Country categorization and relevant weight given to deworming efforts already accomplished

Country category	Country code	Criteria	Effect on estimated years left to reach effective coverage
Effective coverage reached	A	Since 2010 more than 3 years coverage ≥ 75%	Already achieved
Not reached and high coverage	B	Since 2010 less than 3 years coverage ≥ 75% + mean coverage 50% < x < 75%	Regional average duration taken to reach target - *SD*
Not reached and average coverage	C	Since 2010 less than 3 years coverage ≥ 75%, + mean coverage 20% < x < 50%	Regional average duration taken to reach target
Not reached and low coverage	D	Since 2010 less than 3 years coverage ≥ 75%, + mean coverage < 20%	Regional average duration taken to reach target + *SD*

*SD* standard deviation

### Estimated year for achieving five years of effective coverage

To estimate the data in which each endemic country completed five years of effective coverage, we added five years to the year in which the country was estimated to reach effective coverage for the first time. For some endemic countries this date was in the past. For countries in category A, if PC with effective coverage was implemented for more than three years between 2010 and 2017, we subtracted the years they implemented PC with at least 75% coverage from the five total years required by WHO. This took into account deworming efforts made until now. For all countries, the required five years of PC with at least 75% coverage were assumed to have achieved a stable coverage level of 75%.

### Estimated number of children needing annual PC for treatment of STH

To estimate the number of tablets needed, we first calculated the number of children that needed to be covered each year.

To obtain the number of children to be treated annually, we multiplied the number of children requiring treatment in each country by the coverage rate that we expected for each year. The number of children requiring treatment was adjusted according to the rates (%) of national population growth for 2017 from the World Bank. For countries that had not reached effective coverage in 2017, the annual increase in coverage rates was calculated by dividing the coverage gap by the number of years we expected it would take them to reach the target.

### Estimated total number of tablets needed globally

In many STH-endemic countries some areas require two rounds of PC a year and other areas require one. Mupfasoni et al. [6] calculated that, given the differing frequency of PC, the average mean number of tablets needed for each child is 1.6. For this reason, we projected the total number of children to be treated each year and multiplied it by 1.6 to obtain the number of tablets to be distributed every year.

We considered two scenarios:

Scenario 1: Endemic countries, after five years of implementing PC, maintain the frequency of the intervention according to the prevalence at baseline. The number of tablets needed globally increases progressively with the increase in the number of endemic countries achieving effective coverage and the rate of expected population growth in each country.

Scenario 2: Endemic countries, consequent to the reduction in prevalence obtained after five years of PC, reduce the frequency of the intervention as indicated in the WHO decision-tree (Fig. 1).

To calculate the extent of the reduction in the number of tablets needed, we used the study by Mupfasoni et al. [6] that in 2018 examined the 15 countries in all WHO regions to measure changes in STH prevalence after five years of PC and the consequent reduction in need for anthelminthics. The average reduction in STH prevalence was estimated at 70% and the need for anthelminthics was 36%. The reduction of 36% was applied to the number of tablets needed after five years of effective coverage, resulting in a second scenario of future drug needs.

### Independence from drug donations

Scenario 3: We defined a third scenario considering the possibility that countries could support school-based deworming programmes through domestic resources. To do so we divided all STH endemic countries according to the World Bank income groups based on gross national income (GNI) per capita (Table 2).

**Table 2:** World Bank income group (2016) and minimum levels of health financing [9] and expected national self-financing of school deworming

World Bank income group	GNI per capita thresholds (USD)	Number of STH-endemic countries	Minimum health spending per capita (USD 2016)	Minimum government health spending per total health spending (2016)	Expected independency form drug donations
High income	12 056	4	5184	0.782	Independent from 2022 onwards
Upper middle income	3896/12 055	28	461	0.499	Independent from 2022 onwards
Lower middle income	996/3895	35	74	0.284	Progressively independent procurement from 2022 onwards based of comparison of projected health financing levels to minimum levels of 2016
Low income	995	32	38	0.233	Non-independent before 2030

*GNI* gross national income, *STH* soil­transmitted helminthiases, *USD* United States dollar

We chose this method because it models the strategy used by multilateral donors to elaborate the eligibility criteria for donations. Moreover, GNI per capita is a reliable indicator of economic growth, which itself is a known driver of expansion of fiscal space.

We considered that countries in the high-income and upper-middle income groups could become independent from 2022*.* We considered the cost per school child dewormed (USD 0.10–0.20) [4] to be marginal for those high-income and upper-middle income countries whose total health expenditures amount to at least USD 461 per capita.

On the opposite side, countries in the low-income group were attributed full continuation of drug donations because they are the group with the smallest fiscal space and therefore with the most reduced ability to take responsibility for drug procurement.

The lower-middle income group was subjected to a different analysis based on the projections of three health financing indicators: (i) projections for total health spending per capita (2016 US dollars); (ii) projections for government spending as a percentage of total health spending; (iii) projections for total health spending as a percentage of gross domestic product (GDP).

The projected levels of health expenditure were compared with cut-offs derived from the results of a recent (2019) study that summarized the average levels of health financing in 2016 for each World Bank income group [9]. For each indicator, countries with projected levels equal to or greater than the minimum of their income group for 2016 had their drug donations progressively reduced until 2030 according to the matrix indicated in Table 3. The reduction was applied from 2022 based on the reference number of tablets found under scenario 2. We chose to attribute more weight to the variable of government health expenditures per total health expenditures than the other variables in order to demonstrate the importance of public engagement against NTDs. The rationale is that national projected levels of health financing, assumed to be higher than the levels in 2016, can be used as a proxy to model the increased ability of governments to assume responsibility for deworming programmes and therefore to decrease the quantities of drug donations received.

**Table 3:** Criteria for determining the reduction in the number of tablets for lower middle­income countries

National projection equal to or greater than health spending per capita (US$ 2016)	National projection equal to or greater than health spending per GDP (2016)	National projection equal to or greater than government health spending per total health spending (2016)	Attributed effect on drug donations
YES			-20%
YES		YES	-70%
YES	YES		-40%
	YES		-20%
	YES	YES	-70%
		YES	-50%
YES	YES	YES	-80%

*GDP* gross domestic product

## Results

### Estimated year in which endemic countries reach the coverage target and estimated year for completion of five years of PC for treatment of STH

The average time to reach effective coverage in each WHO region was: 5.1 ± 2.5 years in the African Region (AFR); 5.2 ± 3 years in the Region of the Americas (AMR); 7 ± 1 years in the Eastern Mediterranean Region (EMR); 5.2 ± 2.6 years in the European Region (EUR); 4 ± 3 years in the South-East Asia Region (SEAR); and 5.1 ± 2.4 years in the Western Pacific Region (WPR).

Table 4 shows the number of countries in each WHO region for the different categories, the number of years to reach effective coverage and the number of years to reach five years of effective coverage. Globally, 38 endemic countries have reached effective coverage (category A), five countries have not reached effective coverage but have high coverage (category B), 18 countries have not reached effective coverage and are in the average group (category C) and 41 countries fall into the category of countries that have implemented PC with low coverage (category D).

**Table 4:** Number of countries in the four categories (A, effective coverage target reached; B, target not reached yet with high level of coverage during the past seven years; C, target not reached yet with average coverage during the past 7 years; D, target not reached yet with low coverage over the past seven years) and their projected progress to achieve effective coverage

WHO region	Country Code	Number of countries	Mean years left to effective coverage	Mean annual increase in coverage until effective coverage	Years left to the end of 5 years with effective coverage
**African**	A	17	0	NA	0.9
	B	3	2.5	6.8	7.5
	C	6	5.1	8.3	10.1
	D	15	7.6	8.6	12.6
**Americas**	A	7	0	NA	0.7
	B	1	2.2	9.5	7.2
	C	4	5.2	7.3	10.2
	D	13	8.2	8.8	13.2
**Eastern Mediterranean**	A	1	0	NA	2
	C	1	7	6	12
	D	5	8	8.6	13
**European**	A	2	0	NA	0.5
	C	2	5.2	7	10.2
	D	2	7.8	8.9	12.8
**South-East Asia**	A	6	0	NA	1
	B	1	1	10.3	6
	C	1	4	12.8	9
**Western Pacific**	A	5	0	NA	0.8
	C	4	5.1	7.3	10.1
	D	6	7.6	9.5	12.6

*NA* not applicable

**Fig. 2:**
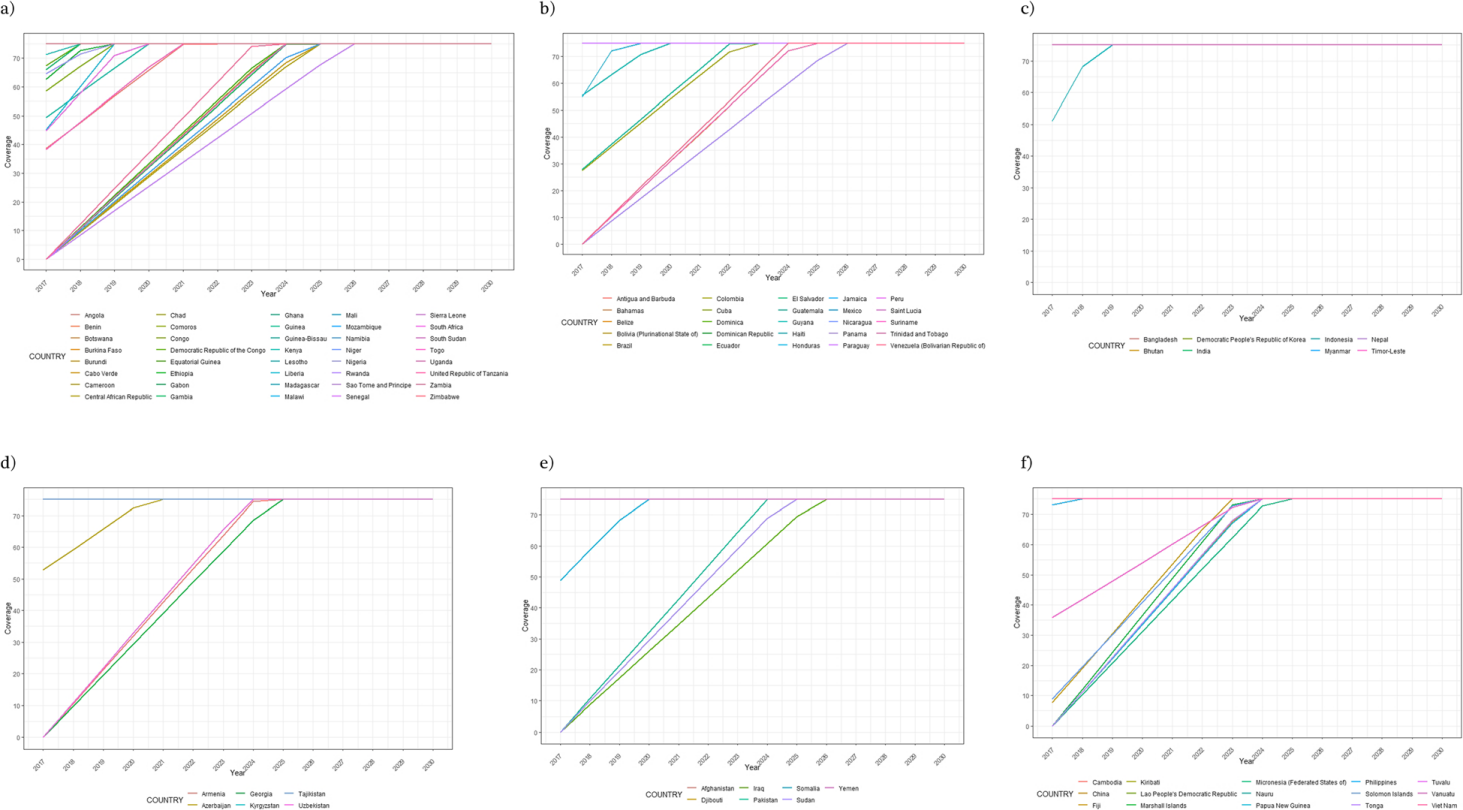
Expected year of achievement of coverage target for STH-endemic countries by WHO region: **a** African Region; **b** Region of the Americas; **c** South-East Asia Region; **d** European Region; **e**; Eastern Mediterranean Region; **f** Western Pacific Region. STH: Soil-transmitted helminthiases; WHO World Health Organization

Figure 2a–f shows the years in which countries will reach effective coverage in the different regions. All countries in AFR, AMR and EMR are expected to reach 75% coverage by 2026; this target is expected to be reached in 2025 by WPR and EUR countries and in 2019 in SEAR countries.

While estimating the year in which each endemic country is expected to reach effective coverage we also estimated annual coverage for each endemic country.

### Estimated number of children needing annual PC for treatment of STH

Table 5 presents the global number of children needing PC for treatment of STH, the total number of children expected to be treated and the global coverage of PC. The total number of SAC in need of deworming is expected to increase from 604 million in 2017 to 754 million in 2030, because of the demographic increase in the population in endemic countries, and the coverage is expected to increase from 61% in 2017 to 75% in 2026.

**Table 5:** Global annual projections of the total number of school-aged children (SAC) requiring preventive chemotherapy (PC) and the total number of children effectively treated

Year	Number of SAC requiring PC (in thousands)	Number of SAC effectively treated (in thousands)	Global coverage (%)
2017	604 085	369 846	61.2
2018	614 252	397 476	64.7
2019	624 630	421 776	67.5
2020	635 224	439 394	69.2
2021	646 039	455 471	70.5
2022	657 082	471 535	71.8
2023	668 357	487 638	73.0
2024	679 870	503 250	74.0
2025	691 627	517 821	74.9
2026	703 635	527 564	75.0
2027	715 899	536 924	75.0
2028	728 426	546 319	75.0
2029	741 222	555 916	75.0
2030	754 294	565 721	75.0

### Estimated total number of tablets needed globally

Table 6 presents the number of tablets needed globally according to scenarios 1 and 2; data for each endemic country are included in the supplementary dataset. In the absence of reduced PC frequency (scenario 1), the need for anthelminthics is expected to reach approximately 900 million tablets in 2030. However, should countries reduce the frequency of the intervention according to the WHO decision-tree (Fig. 1, scenario 2), the need for anthelminthics will be reduced to 460 million in 2030.

**Table 6:** Global annual projections of numbers of tablets needed under scenarios 1, 2 and 3

Year	Scenario 1 Total no. of tablets (in thousands) with no consideration of impact of deworming programmes on prevalence reduction	Scenario 2 Total no. of tablets (in thousands) with 36% reduction in no. of tablets needed following prevalence reduction every five years of PC implementation with effective coverage	Scenario 3 Total no. of tablets (in thousands) with progressive phasing out of drug donations. Results of univariate sensitivity analysis in parenthesis.
2017	591 751	546 603	546 603
2018	635 958	585 432	585 432
2019	674 838	534 948	534 948
2020	703 027	561 139	561 139
2021	728 749	584 828	584 828
2022	754 452	577 459	370 224 (284 943–450 908)
2023	780 215	594 601	377 245 (290 609–461 146)
2024	805 194	540 395	325 958 (267 142–402 721)
2025	828 508	559 632	335 943 (276 153–415 993)
2026	844 095	551 456	333 383 (272 740–393 256)
2027	859 071	489 806	300 247 (240 639–348 937)
2028	874 104	496 910	304 582 (244 205–354 161)
2029	889 459	470 456	288 683 (228 751–338 970)
2030	905 146	460 526	231 507 (215 746–331 983)

*PC* preventive chemotherapy

### Independence from drug donations

In scenario 3, we assumed that countries in the high-income and upper-middle income groups of the World Bank could become progressively independent from drug donations by using domestic resources to procure the anthelminthics needed in school-based deworming programmes.

Table 2 shows the number of endemic countries classified by World Bank income group.

In total:

- 4 endemic countries were classified as high-income and 28 as upper-midle income and are therefore expected to become independent in supporting PC for treatment of STH from 2022;
- 35 endemic countries were classified as lower-middle income and are therefore expected to become progressively independent between 2022 and 2030; and
- 32 endemic countries were classified as low-income and are therefore expected to continue to receive full external support until 2030.

**Fig. 3:**
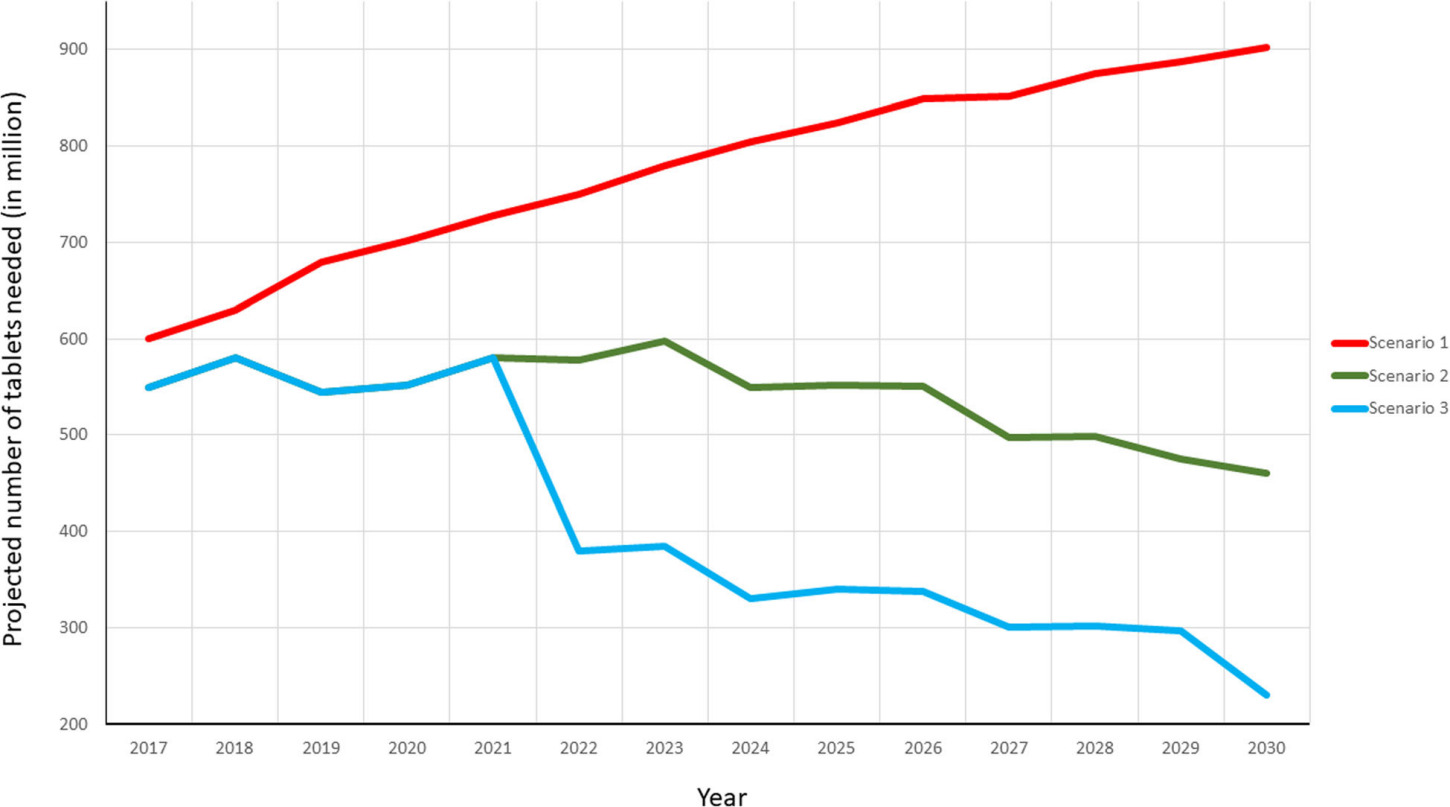
Summary graph of the three scenarios of projections for total number of tablets donated annually until 2030. Three scenarios are presented: Scenario 1: Endemic countries would continue to increase coverage and maintain the same frequency of intervention. Scenario 2: Endemic countries would reduce the frequency of intervention when the STH prevalence is reduced, according to the WHO decision-tree. Scenario 3: Some endemic countries would sustain the cost of the deworming programme, depending on the levels of development

Under scenario 3 we observed that the number of “donated” tablets needed globally decreases by 39.9% by 2025 and by 49.7% by 2030 compared with the number of tablets estimated under scenario 2. Compared with the forecast obtained under scenario 1, this change represents a 59.4% reduction by 2025 and a 74.4% reduction by 2030. The WHO African and South-East Asia regions are the main regions that will continue to need drug donations until at least 2030. This can be explained by the predominance of low-income and lower-middle income countries in these regions. Figure 3 compares the estimated number of tablets needed according to the three scenarios.

### Sensitivity analysis

As reported in Table 6, a 20% univariate sensitivity analysis was conducted to evaluate the variability of the results when national projections for the three health financing indicators are varied by 20%. We created an interval within which we believe the numbers found under scenario 3 are likely to vary.

The numbers of tablets needed in scenarios 2 and 3 reduce to 50.6% by 2025 and to 53.1% by 2030 if we assume that national projections on health spending are 20% higher than those reported by the Institute for Health Metrics and Evaluation. These numbers reduce further to 25.6% by 2025 and to 27.9% by 2030 in a scenario in which national health financing projections are 20% less than the findings of the Institute for Health Metrics and Evaluation.

In scenarios 1 and 3, the numbers of tablets needed are reduced to 66.6% by 2025 and to 76.1% by 2030 if we assume national health spending will be 20% higher than those projected by the Institute for Health Metrics and Evaluation. They reduce to 49.7% by 2025 and to 63.3% by 2030 should the national health financing be 20% less than the projections of the Institute for Health Metrics and Evaluation.

## Discussion

Our findings on the effective coverage target are promising: by 2030 all endemic countries will have reached the target of implementing five consecutive years of PC with effective coverage. We have demonstrated that STH morbidity reduces significantly after 5 years of PC [10]. We therefore believe that achievement of target 3.3 of Sustainable Development Goal 3 (“Ensure healthy lives and promote well-being for all at all ages”), which extends Millennium Development Goal 3 beyond HIV, TB and malaria to “end the epidemics” of NTDs by 2030 [11] is feasible for STH.

The reduction in the total number of tablets donated by pharmaceutical companies as estimated in this study is relevant, but our estimates are conservative for the following reasons:

- Our method for defining when a country reached effective coverage (based on average levels over 7 years) is stricter than that normally used by WHO, which considers that the effective coverage is reached when a country reaches this level for the first time; and
- Rapid economic development may result in countries shifting from the low-income group to the lower-middle income group or from lower-middle income to upper-middle income countries.

We therefore consider that the number of donated tablets needed may reduce more rapidly than estimated.

To measure countries’ ability to implement deworming programmes independently, we took into account variables related to their level of development and measure of welfare (World Bank income groups) and to health financing. GNI per capita, based on a determination of income group thresholds, has limitations as an indicator of wealth and development. GNI, calculated as GDP plus net receipts from abroad of wages and salaries and of property income plus net taxes and subsidies receivable from abroad [12], does not capture inequalities and income distribution (OECD, 2012). This idea was first explored by the economist Amartya Sen who showed that societies’ level of well-being is determined not only by its level of net income per capita but also by how that income is used and distributed [13]. Moreover, the concept of economic development as measured by traditional instruments (GDP, GNI) fails to acknowledge the choices of how income is used and can therefore bias estimates of the magnitude of efforts by countries to increase the well-being of their populations. GNI per capita may be a good theoretical tool for measuring countries’ ability to finance deworming programmes independently, but it is insufficient on its own. Other instruments such as the human development index (HDI) might also be a good indicator of exogenous elements, such as political will, which are likely to influence how priorities are identified when States elaborate annual budgets and plan national public health programmes [14]. The division of countries into no reduction, progressive reduction and total discontinuation groups for drug donations according to the World Bank income groups is thus driven only by the necessity to rely on available estimates of internationally comparable numbers. The additional consideration of health financing indicators, at least for the group of low-middle income countries, is also an attempt to include and “control” for other factors that have an impact on NTDs, in addition to GNI per capita.

A strategy to progress phasing out of drug donations should nevertheless be carefully organized to ensure that programmes are not interrupted. Additional elements that influence a country’s ability to become independent in the fight against NTDs should be taken into consideration.

We were unable to include the following elements in our analysis:

- The possible contribution of improvements in water management and sanitation systems because of the difficulty in predicting investments in this sector; however, substantial global improvement in access to water and sanitation is expected to result from efforts to reach Sustainable Development Goal (Goal 6: Clean water and sanitation) [15];
- The expected improvement in the quality of education (Goal 4: Quality education), especially if targeted towards maternal education, which is known to accelerate elimination of NTDs [16]; however, it is very difficult to quantify the impact of girls’ education today in reducing the risky behaviours among and reinfection of children tomorrow; and
- the expected progress in elimination of poverty (Goal 1: No poverty) that will entail substantial reduction in STH endemicity [17].

Again, if all these goals are reached the reduction in the need for donated drugs to control STH could be greater than estimated.

## Conclusions

Coverage of PC for treatment of STH has increased in recent years, yielding benefits for control of morbidity tomorrow, important increases in adults’ productivity and high returns on public investment. The prospect of progressively reducing drug donations for PC creates a case for countries to prioritize the fight against NTDs. Our study found that during 2020–2030, all countries in which STH are endemic are expected to reach the effective coverage target and that improved STH epidemiology will reduce the number of tablets needed to implement PC globally. Moreover, thanks to the reduction of the total cost of PC associated with these epidemiological improvements, the magnitude of required deworming programmes will also shrink considerably, making it more affordable for endemic countries to assume implementation of PC. Low-income countries will likely not become independent in implementing PC because of the current low level of development and the limited improvements expected in the next 10 years. Therefore, while donations of anthelminthic medicines will continue to be necessary, the number of donated anthelmintic tablets needed will reduce by 74.4%.

We should not forget that the permanent elimination of STH is feasible only with the elimination of environmental contamination, which can be achieved by drastically improving the coverage of sanitation and promoting behavioural change.

## Data Availability

All supporting data are made available.

## Abbreviations

AFR: WHO African Region
AMR: WHO Region of the Americas
EMR: WHO Region for the Eastern Mediterranean
GDP: Gross domestic product
GNI: Gross national income
HDI: Human development index
NTD: Neglected tropical disease
OECD: Organisation for Economic Co­operation and Development
PC: Preventive chemotherapy
SAC: School-aged children
SEAR: WHO Region for South­East Asia
STH: Soil­transmitted helminthiases
WHO: World Health Organization
WPR: WHO Region for the Western Pacific
